# Mechanistic investigation of the hepatotoxicity and potential carcinogenicity of microcystin-LR, -LA, and -RR in the HepaRG cell line model

**DOI:** 10.1007/s00204-026-04458-0

**Published:** 2026-06-08

**Authors:** Enzo Zini Moreira Silva, Sahra Kiessig, Ilinca Suciu, Carolina Mathias, Marize de Lourdes Marzo-Solano, Cynthia Bomfim Pestana, Philip Marx-Stoelting, Daniela Morais Leme

**Affiliations:** 1 Department of Genetics, Federal University of Paraná (UFPR), Curitiba, PR Brazil; 2 German Federal Institute for Risk Assessment (BfR), Berlin, Germany

**Keywords:** Microcystins, HepaRG cell line, PP2A activity, Cell proliferation, Pathway-focused PCR array profiling

## Abstract

Microcystins (MCs) are cyanobacterial toxins found in water and food and are suspected to pose significant health risks, particularly to the liver. To gain insight into the mechanisms of hepatotoxicity and the potential carcinogenicity of MCs, this study investigated the effects of MC-LR, -LA, and -RR (congeners differing in cellular uptake and detoxification efficiency) on PP2A activity, cell proliferation, and gene expression profiling focused on toxicity pathways and carcinogenesis in HepaRG cells. The three MCs similarly inhibited PP2A activity; however, only MC-LR and -LA significantly increased cell proliferation. Gene expression profiles (mRNA) revealed strong, similar treatment effects induced by MC-LR and -LA (R2 = 0.66), whereas -RR affected fewer genes, showing weak correlations with the other two congeners (-LRx-RR – R^2^ = 0.31; -LAx-RR – R^2^ = 0.06). Moreover, MC-LR and -LA, but not -RR, affected several liver-enriched toxicity pathways, which correlate most closely to hepatocellular/liver carcinoma, cholestasis, and fibrosis. All congeners altered carcinogenesis-related miRNA expression, but no consistent pattern was observed among them. Overall, the present findings reinforce the congener-specific toxicity of MCs, likely driven by differences in toxicokinetics among congeners. Furthermore, the findings indicate that MC-induced liver effects also involve PP2A-independent mechanisms, as MC-LR/-LA and -RR elicited markedly different biological responses despite comparable PP2A inhibition. Finally, the study demonstrates the applicability of the NAM-based approach for evaluating liver effects (hepatocytes) in the MC family and, as structurally different congeners were investigated, provides data that could be used for read-across and grouping.

**Supplementary Information:**

The online version contains supplementary material available at https://doi.org/10.1007/s00204-026-04458-0.

## Introduction

Microcystins (MCs) constitute a family of structurally related cyclic oligopeptide toxins produced by various cyanobacterial species. They are widely distributed in aquatic environments, and their occurrence increases substantially during algal blooms. Consequently, drinking water is the most likely route of exposure to MCs, and the safety limit is defined as 1 µg/L. However, due to high uncertainty arising from data gaps on MC hazard properties, this is a provisional guideline value (WHO 2020, 2022). In addition to water, the occurrence of MCs in food, especially fish, mussels, and shellfish, may pose human health risks, and a tolerable daily intake (TDI) of 0.04 μg/kg body weight was proposed for chronic exposure (WHO 2022). However, this value is similarly derived with high uncertainty and is based primarily on data from MC-LR (Testai et al. 2016; WHO 2022).

Structurally, the MCs share the general formula cyclo-(DAla^1^-X^2^-D-MeAsp^3^-Z^4^-Adda^5^-D-Glu^6^-Mdha^7^), in which X and Z are variable amino acids. Substitutions at these variable amino acid positions, as well as alterations at other amino acid positions, result in a wide diversity of congeners, with 310 so far identified. These subtle structural variations are also responsible for differences in toxicity and potency among congeners. Elucidating such differences is important to improve MC human risk assessment. For instance, the proposed toxicity equivalent factor concept may over- or underestimate the toxicodynamic capacity of a cyanobacterial bloom if the most toxic congener is incorrectly identified (Altaner et al. 2020; Santori et al. 2020). Despite data gaps, MC-LR is considered the most toxic congener and is classified as possibly carcinogenic to humans (Group 2B, IARC) based on in vivo tumor promotion activity (IARC, 2010).

The health effects of MCs are primarily associated with liver dysfunction, as cellular uptake of these toxins is mediated by organic anion-transporting polypeptide (OATP) transporters, which are highly expressed in liver cells (Jetter and Kullak-Ublick, 2020). The most accepted mechanism of MC toxicity in the liver involves inhibition of the protein phosphatase 2A (PP2A) (Testai et al. 2016; WHO  2022). The PP2A inhibition leads to downstream protein hyperphosphorylation, thereby reversing the actions of protein kinases that regulate a wide range of cellular processes (Yin et al. 2025). Despite that, the mechanistic basis for the wide variety of toxic effects of MCs in the liver, including the well-studied MC-LR, remains unclear. Furthermore, recent studies have shown that MCs may have other regulatory proteins as molecular targets, which could help explain the complexity of their biological responses in the liver (Yang et al. 2023; Lynch et al. 2023; Ikumawoyi et al. 2024; Yin et al. 2025). Epigenetic effects potentially caused by MCs, such as dysregulation of non-coding RNAs (*e.g.*, miRNAs), are also not yet fully explored, while their role in diseases, including cancer, has become increasingly emphasized (Chen et al. 2018; Feng et al. 2020; Florczyk et al. 2021).

Human in vitro models have been proposed to gain knowledge on congener-specific toxicity and to advance MC risk assessment, as the limited availability of high-purity MCs constrains in vivo data generation (Hill et al. 2022; Pestana et al. 2025). Furthermore, human cell models enhance biological relevance, reducing uncertainties associated with interspecies differences. For instance, rodents do not accurately reflect human tissue OATP expression as well as substrate specificity and transport efficiency for specific congeners (Santori et al. 2020). The HepaRG cell line is a bipotent human progenitor cell line derived from a liver tumor of a female patient. These cells can be differentiated into hepatocyte- and cholangiocyte-like cells, and retain characteristics similar to mature human hepatocytes, including the expression of the major P450s (Gripon et al. 2002; Aninat et al. 2006; Kuna et al. 2018; Zheng et al. 2021; Donato et al. 2022). These features have made the HepaRG cell line extremely attractive for advancing chemical safety assessment, and several New Approach Methodologies (NAMs) using this model have recently been proposed for liver toxicity and carcinogenicity investigations (Gutmann et al. 2023; Sherer et al. 2025; Jochum et al. 2025; Saleh et al. 2026). Furthermore, HepaRG, unlike other cell lines, e.g., HepG2, presents appropriate OATPs’ expression levels, a key feature for in vitro investigations of MCs’ toxicity.

In this study, three MC congeners differing in affinity for OATP1B1 and OATP1B3 and detoxication efficiency (MC-LR, -LA, and -RR) (Santori et al. 2020; Chowdhury et al. 2024) were studied in the HepaRG model at the toxicodynamic level to gain mechanistic insights into their hepatotoxic and potential carcinogenic effects. Toxicity at the individual level is necessary to characterize the risk of chemical mixtures, a key for the MC family as environmental occurrences typically involve multiple congeners. Therefore, the toxicodynamic knowledge gained for individual congeners can further support mixture assessment of MCs.

## Material and methods

### Tested microcystins

MC-LR (CAS No. 101043–37-2; ALX-350–012), MC-LA (CAS No. 96180–79-9; ALX-350–096), and MC-RR (CAS No. 111755–37-4; ALX-350–043) were obtained from Enzo Lifescience, all of them with > 95% of purity. Stock solutions (1 mg/ml) of each MC were prepared in dimethyl sulfoxide (DMSO), and working solutions were prepared by diluting the stock solutions into the treatment media for HepaRG cells (described below), maintaining a final DMSO concentration of 0.5% (v/v).

### Cell culture

The HepaRG cell line was obtained from Biopredic International (Saint Grégoire, France), and the cells were cultured according to the protocol described by Karaca et al. (2023). Briefly, HepaRG cells were cultivated in 96-well (9 × 10^3^ cells/well) or 24-well plates (3.6 × 10^4^ cells/well) (Greiner) in proliferation medium ((William’s Medium E with 2 mM glutamine (PAN-Biotech, Aidenbach, Germany), 10% (v/v) of fetal bovine serum (FBS Good Forte EU approved; PAN-Biotech, Aidenbach, Germany), 100 U/ml penicillin and 100 μg/ml streptomycin (Capricorn Scientific, Ebsdorfergrund, Germany), 0.05% (v/v) human insulin (PAA Laboratories GmbH, Pasching, Austria), and 50 μM hydrocortisone hemisuccinate (Sigma-Aldrich, St. Louis, MO, USA)) for 14 days at 37 °C, 5% CO_2_ and in humidified atmosphere. Afterward, the medium was exchanged with the transition medium (proliferation medium containing 1% DMSO). After another 24 h, the transition medium was exchanged with differentiation medium (proliferation medium containing 1.7% DMSO) to promote cell differentiation over a 14-day incubation period. After 29 days, HepaRG cells are fully differentiated. Before exposure to test agents, the cells are stabilized in treatment medium (proliferation medium containing 2% FBS and 0.5% DMSO) for 24 h.

### Exposure conditions

Cytotoxicity assays were performed in 96-well plates using eight concentrations of each MC: 0.04, 0.08, 0.156, 0.312, 0.625, 1.25, 2.5, and 5 μM. Cell proliferation and PP2A activity assays were performed in 24-well plates and with three concentrations of the MCs: 0.3, 1.1, and 2.5 μM. Gene expression was analyzed in HepaRG cells exposed to a single concentration of each MC (2.5 μM). 24 h exposure was defined as the exposure time for all markers evaluated. All experiments were performed in three technical replicates (n = 3) and three independent experiments (N = 3), except for the gene expression analysis, which did not include technical replicates due to the qPCR array configuration. HepaRG cells cultured in treatment medium were used as a negative control (NC). Triton X-100 at 0.1% (v/v), colchicine (COL – Sigma, 95% purity) at 0.2 µM, and okadaic acid (OA – Enzo Lifescience, 98% purity) at 62.5 nM were used as positive controls (PCs) for the cytotoxicity, cell proliferation, and PP2A activity, respectively.

### Cytotoxicity assay

WST-1 (Sigma-Aldrich, St. Louis, MO, USA) and Neutral Red (NR) were used as cell viability indicator dyes in the cytotoxicity assay, as described by Karaca et al. (2023). After the MCs’ exposure, HepaRG cells were washed with PBS, incubated with WST-1 (100 μl/well) for 30 min at 37 °C with 5% CO_2_, and the absorbance for this cell viability marker was measured at 450 nm using the Infinite M200 Pro plate reader spectrophotometer (Tecan). Then, the cells were washed again with PBS and incubated with the NR solution (0.005%) for 2 h. For NR, fluorescence was measured at 530 nm excitation and 645 nm emission using the Tecan instrument. Cell viability was calculated relative to the NC (100% viability).

### Cell proliferation assay

A flow cytometry-based propidium iodide (PI) cell cycle assay was used to evaluate the proliferative effect of MCs on differentiated HepaRG cells. MCs-exposed HepaRG cells were harvested using trypsin (0.025%), washed 2 × with PBS, and incubated in ethanol 70% (fixative solution) for 30 min at 4ºC. After fixation, cells were washed once with PBS, resuspended in staining solution (50 μg/ml PI and 100 μg/ml RNase A in PBS), and incubated at room temperature (RT) for 30 min. The BD Accuri C6 flow cytometry instrument was used for data acquisition, and the FlowJo software for data analysis. The cell cycle phases were determined by DNA content, and cells in the S and G2/M phases were used to evaluate the effect on cell proliferation. Significant increases in the NC (p < 0.05, one-way ANOVA followed by Tukey’s multiple comparisons test) were considered a positive response in cell proliferation.

### PP2A activity assay

The effects of MCs on PP2A activity were evaluated by the malachite green assay using the Ser/Thr Phosphatase Assay Kit 1 (K-R-pT-I-R-R) (Merck, Cat. No. 17–127), according to the manufacturer’s instructions. HepaRG cells exposed to MCs were harvested using Tris–EDTA buffer (100 mM Tris, 5 mM EDTA) containing 0.1% ß-Mercaptoethanol and 0.5 mg/ml bovine serum albumin (BSA) at pH 7.2, and lysed by sonication in an ultrasonic bath for 15 s. The solutions were centrifuged (30 min, 10,000 g, 4 °C), and desalting columns (PD SpinTrap G-25—Cytiva, Cat.: 28,918,004) were used to remove free phosphate from the samples. The samples were mixed with phosphopeptide in a 96-well plate and incubated for 30 min at RT for the reaction. The malachite green and the stop solution were added simultaneously to the plate, which was incubated for another 30 min at RT and protected from light. The absorbance was measured at 650 nm using the Tecan instrument. For data normalization, total protein content was quantified in each sample using the Bradford assay (ROTI®Quant 5X, Carl Roth, Cat. No. K015.2). 20 μL of each sample was mixed with 180 μL of 1 × RotiQuant solution in a 96-well plate, and the absorbance was measured at 595 nm using the Tecan instrument. The standard curve and R^2^ were calculated to normalize malachite green results. Normalized data were statistically analyzed using one-way ANOVA followed by Tukey’s multiple comparisons test to identify significant differences related to NC (p < 0.05) in the decrease of PP2A activity.

### Pathway-focused PCR array profiling analysis

Changes in protein-coding genes (mRNA) and miRNAs were evaluated using the following RT2 Profiler™ PCR Arrays (QIAGEN): Human Molecular Toxicology PathwayFinder 384HT (GeneGlobe ID: PAHS-3401Z), Human Cancer PathwayFinder X-Large Focus (miScript 384HC) (GeneGlobe ID: YAHS-402Z) (Tables 1S and 2S). Arrays, as well as the kits described below, were used according to QIAGEN’s instructions, the supplier of all reagents used for this analysis. For mRNA extraction and cDNA synthesis, the RNeasy Mini Kit (Cat. No. 74104) and the RT2 First Strand Kit (Cat. No. 330404) were used, respectively. miRNA was extracted using the miRNeasy Micro Kit (Cat. No. 217084), and for the cDNA, the miRCURY LNA RT Kit (Cat No. 339340) was used. The RT2 SYBR Green ROX qPCR Mastermix (Cat No. 330529) and miRCURY LNA SYBR Green PCR Kit (Cat No. 339345) were used for the quantitative reverse transcription polymerase chain reaction (RT-qPCR) with cDNA derived from mRNA and miRNA samples, respectively. The mRNA array contains primer assays for five endogenous reference genes (*ACTB*, *B2M*, *GAPDH*, *HPRT1*, *RPLP0*) and nine additional assay controls (genomic DNA control, reverse transcription control, and positive PCR control) to monitor reaction performance. For the miRNA analysis, the UniSp6 probe was used to ensure qPCR quality. The equipment used for qPCR analysis was an ABI 7900HT Fast Real-Time PCR (Applied Biosystems), and data were extracted from the software ABI 7900 v241.

To identify differentially expressed genes (DEGs) and differentially expressed miRNAs (DE miRNAs), the GeneGlobe Dashboard™ (QIAGEN) was used. The cycle threshold (C_t_) cut-off was set to 35. The arithmetic Ct mean of the endogenous genes was used to normalize the mRNA data, and the global Ct mean was used for the miRNA data. The multiple-test correction was performed using the Benjamini–Hochberg method for mRNA and miRNA. Genes that presented an absolute log2 fold-change ≥ 1 (up or downregulation) compared to the NC and with adjusted *p*-value < 0.05 were considered differentially expressed (Table 3S-8S). The list of DEGs was used for pathway and network analysis using QIAGEN Ingenuity Pathway Analysis (IPA), and an experimental p-value < 0.1 was used as the cut-off for pathway significance (mRNA dataset).

An exploratory analysis was conducted in R (v4.4.0) using raw qPCR data normalized via the ΔΔCt method, applied to individual replicates instead of group means. Replicate similarity was assessed using pairwise Pearson correlation of ΔCt values, with all correlation coefficients exceeding 0.9, indicating high consistency across replicates and no apparent technical outliers. These results were further supported by principal component analysis (PCA), performed using the prcomp() function from the stats package.

Gene expression changes across MCs’ treatments (relative to control) were visualized using heatmaps generated with the ComplexHeatmap package (v2.22.0). To investigate functional relationships, flow diagrams were created for each treatment condition using the networkD3 package (v0.4.1), illustrating connections between disease and toxicity pathways. This analysis was based on the "Diseases or Functions Annotations" (DFA) and "Ingenuity Toxicity Lists" (ITL) categories extracted from IPA. Each significant DFA term was manually assigned to a corresponding disease category, while ITL terms were grouped as toxicity pathways. In cases where the DFA and ITL categories overlapped, naming was harmonized accordingly. Each shared DEG was represented as a link between the two categories, and for clarity, these genes were also included as intermediate nodes.

Additionally, to investigate regulatory relations among miRNAs and mRNAs, the R package “multiMiR” (Ru et al. 2014) was used. These potential regulatory interactions were determined between DE miRNAs and DE mRNAs; in this context, only experimentally validated data and negative expression correlations between mRNA and miRNA were considered (Hausser and Zavolan 2014). These parameters are part of the definition of competing endogenous RNA (ceRNA) networks, which play a key role in the regulation of gene expression.

## Results

### Cytotoxicity, cell proliferation, and PP2A activity

**Fig. 1:**
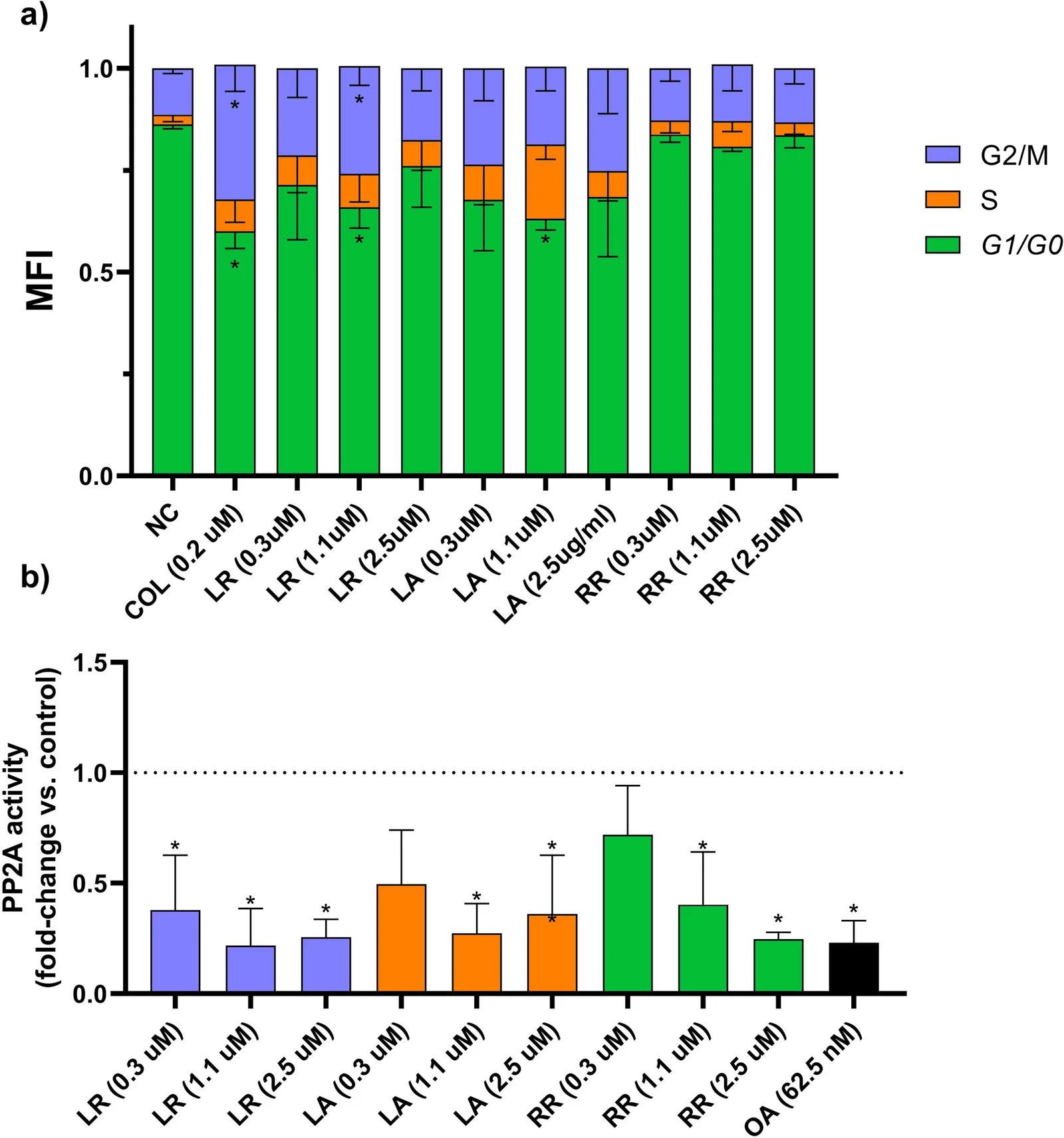
**a** Results of cell proliferation evaluated by cell cycle analysis (G1/G0, S, G2/M) using propidium iodide (PI) in flow cytometry. **b** Results of the PP2A activity evaluated by the malachite green phosphate assay. Colchicine (COL) and okadaic acid (OA) were used as positive controls in cell proliferation and PP2A activity assays, respectively. Flow cytometry data are expressed as mean ± SD of the median fluorescence intensity (MFI) of PI relative to the negative control (NC). PP2A data are expressed as mean ± SD of the fold-change relative to NC. *Statistically significant differences related to NC (*p* < 0.05)

The tested MCs exhibited concentration-dependent cytotoxic effects. The responses to MC-RR were similar for both cell viability markers, whereas stronger cytotoxic effects were observed in the WST-1 assay with MC-LR and -LA (Fig. S1). Regarding cell proliferation, MC-LR and -LA showed significant increase in cells at S + G2/M at 1.1 µM and a significant decrease in cells at G1/0 when compared to the NC, indicating that MC-LR and -LA probably cause proliferative effects (Fig. 1a). The results of the malachite green phosphate assay for PP2A showed that the three MCs were capable of significantly inhibiting the PP2A activity at the highest tested concentrations (1.1 and 2.5 μM), and also at the lowest concentration for MC-LR (0.3 µM). At 2.5 μM, the MC-LR, -LA, and -RR have, respectively, a fold inhibition of 0.76, 0.71, and 0.66.

### Gene expression: pathway-focused PCR array profiling

#### Differentially expressed genes (DEGs)

**Fig. 2:**
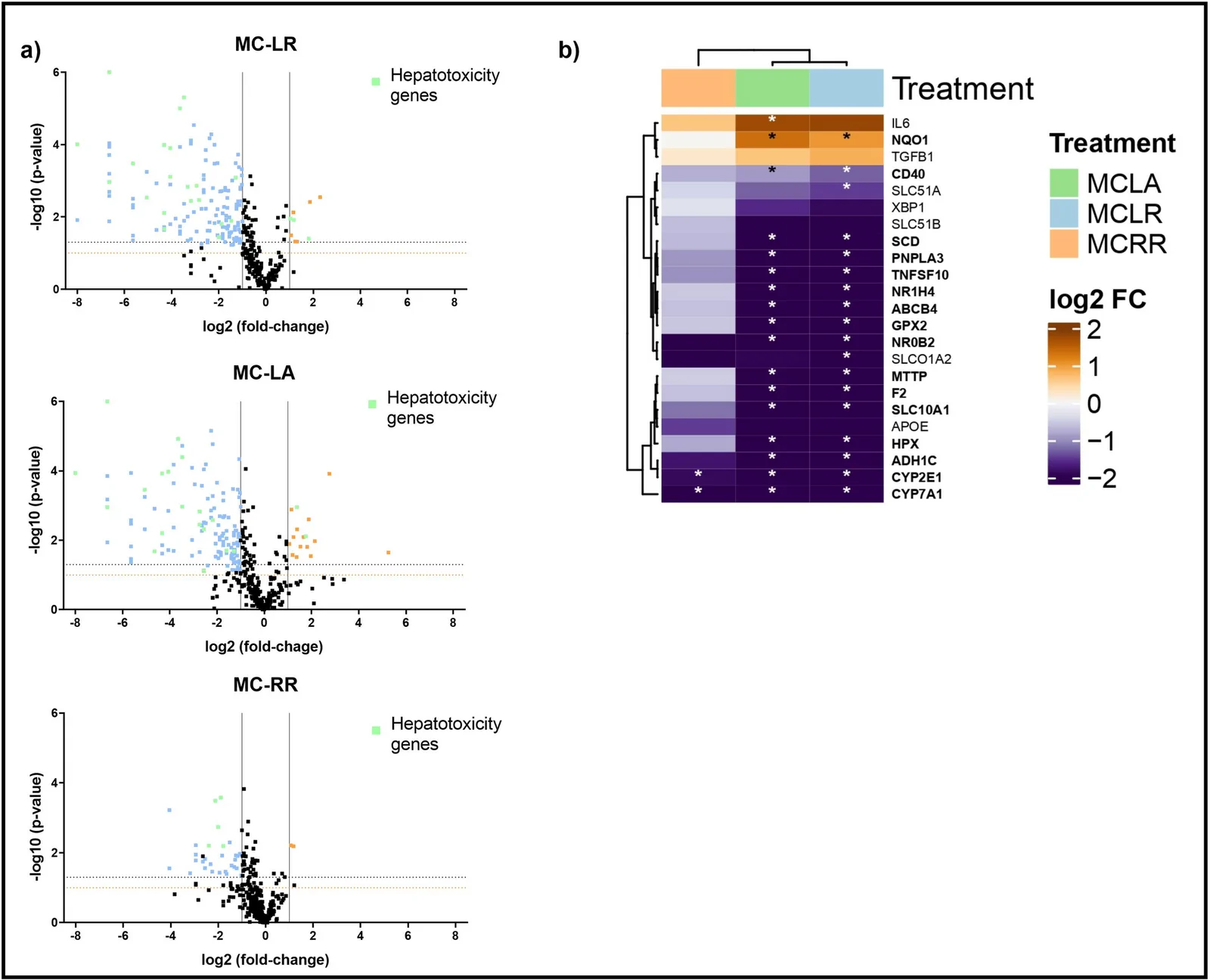
Hepatotoxicity-related genes from the employed toxicity gene panel. **a** Volcano plot showing the gene expression data obtained from HepaRG cells exposed (24 h) to MC-LR, MC-LA, and MC-RR (2.5 µM). Light green-dot = genes related to hepatotoxicity, black-line = adjusted *p*-value < 0.05, red line = adjusted p-value < 0.1. **b** heatmap of hepatotoxicity-related genes, * adjusted *p*-value < 0.05

The PCA revealed that MC-LR and -LA showed stronger, similar treatment effects on the expression of protein-coding genes (mRNA), whereas -RR showed treatment effects closer to the NC (Fig. S2). The strong correlation between MC-LR and -LA (R^2^ = 0.66) is also evident from the linear regression analysis, while -RR showed a weaker correlation with both congeners (R^2^ = 0.31 for MC-LR and 0.06 for MC-LA) (Fig. S3). In terms of the number of differentially expressed genes (DEGs), MC-LR and -LA caused perturbations in the expression of a higher number of genes related to hepatotoxicity and carcinogenicity than -RR (Fig. 2a, 3a). The expression patterns of the affected genes were predominantly downregulated, including by -RR, which showed fewer DEGs (Fig. 2b, 3b). However, the extent of downregulation in genes associated with these two endpoints was more pronounced and closely aligned in MC-LR and -LA, suggesting that these congeners may exert stronger effects in the liver than -RR. Heatmaps showing log2 fold changes and z-scores for the overall gene panel used and treatment conditions are presented in the supplementary material (Fig. S4-S5).

**Fig. 3:**
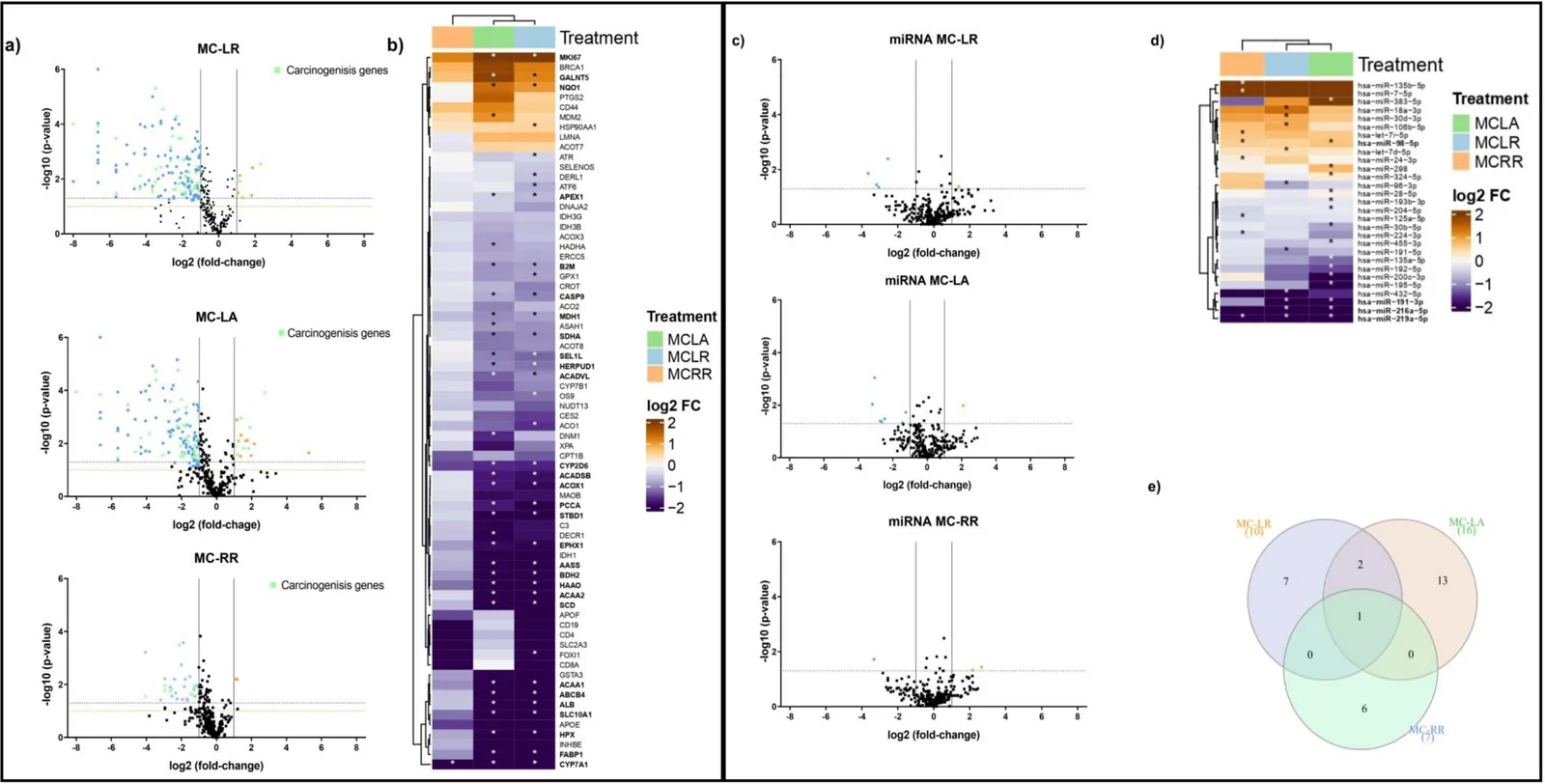
Carcinogenesis-related genes from the employed toxicity gene panel. **a** mRNA volcano plot of cells exposed to MC-LR, MC-LA, and MC-RR (2.5 µM) for 24 h, light green-dot = genes related to carcinogenesis; black-line—adjusted *p*-value = 0.05, red line—adjusted *p*-value = 0.1. **b** heatmap of carcinogenesis-related genes. **c** miRNA volcano plot of cells exposed to MC-LR, MC-LA, and MC-RR (2.5 µM) for 24 h. **d** Heatmap of carcinogenesis-related miRNA. **e** Venn diagram of differentially expressed miRNA of each tested MC. * adjusted *p*-value < 0.05

Regarding miRNA, the three MCs modulated the expression of miRNAs associated with carcinogenesis; however, in contrast to mRNA, there was very limited concordance in the specific miRNAs deregulated by each MC. The miR-219a-5p was the only miRNA commonly deregulated across all MC treatments (Fig. 3d-e). A global heatmap showing the expression pattern of the control and tested MC is presented in Fig. S6.

#### Pathway enrichment analysis: ingenuity pathway analysis (IPA)

**Fig. 4:**
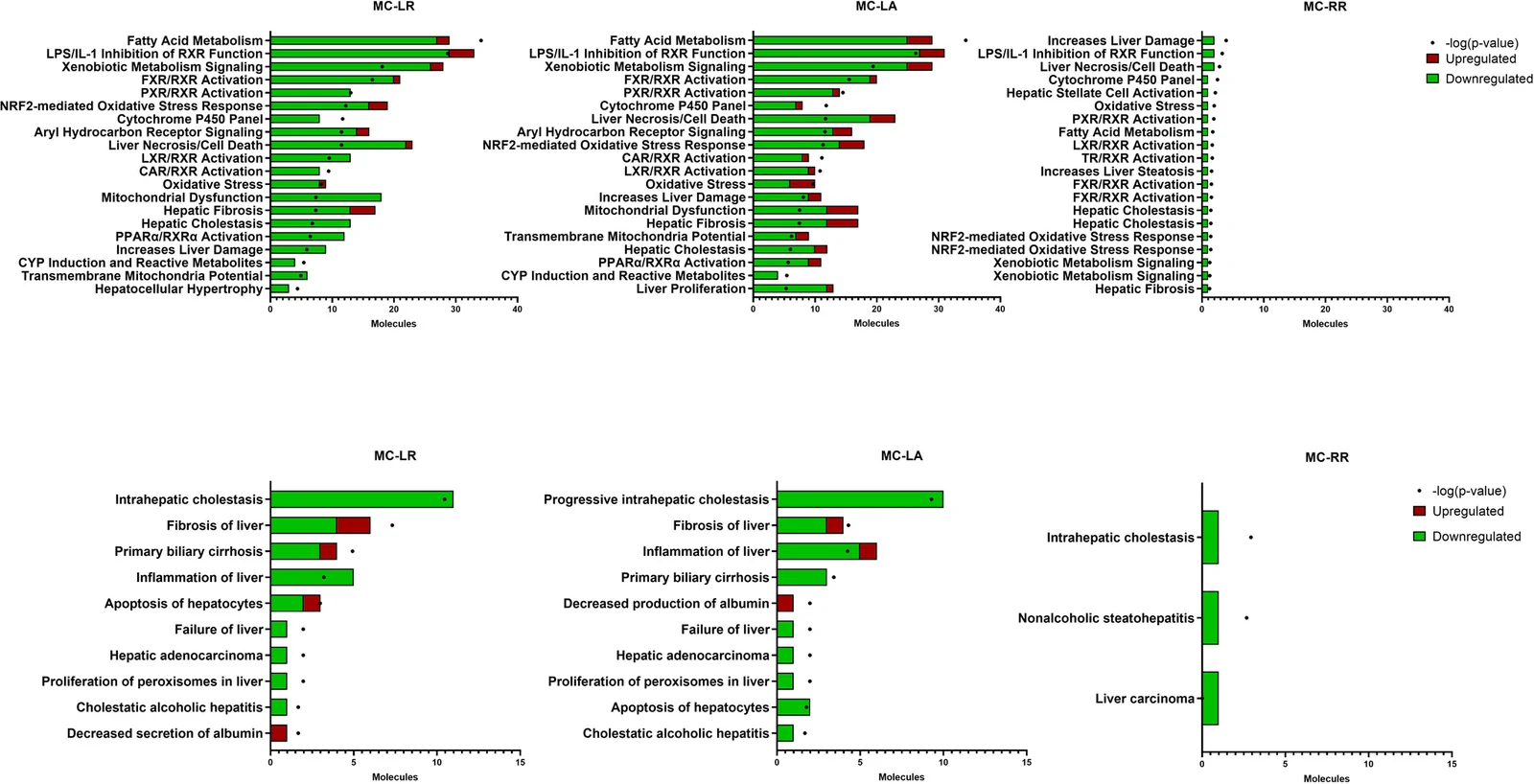
The top 20 toxicity pathways and top 10 diseases/function annotations predicted to be affected for each MC by the IPA. The pathways were ranked in ascending order of –log (*p*-value); the x-axis represents the number of up- and down-regulated genes and *p*-values

The top 20 enriched toxicity pathways and top 10 enriched disease pathways identified by IPA are shown in Fig. 4. The DEGs induced by MC-LR and -LA were highly enriched in pathways of xenobiotic metabolism, nuclear receptor signaling, mitochondrial/metabolic dysfunction, and oxidative stress/cellular defense. The DEG signatures and enriched liver-toxicity pathways link with liver dysfunctions, mostly cholestasis, fibrosis, and hepatocellular/liver carcinoma. For MC-RR, although perturbations also reached statistical significance in liver toxicity pathways, the limited gene overlap (*i.e.*, 1–2 DEG enriched per toxicity pathway or disease/function annotation) reduced the robustness of the inferred associations with such liver injuries and diseases.

#### Correlation of disease/function annotation relationship and toxicity pathways

**Fig. 5:**
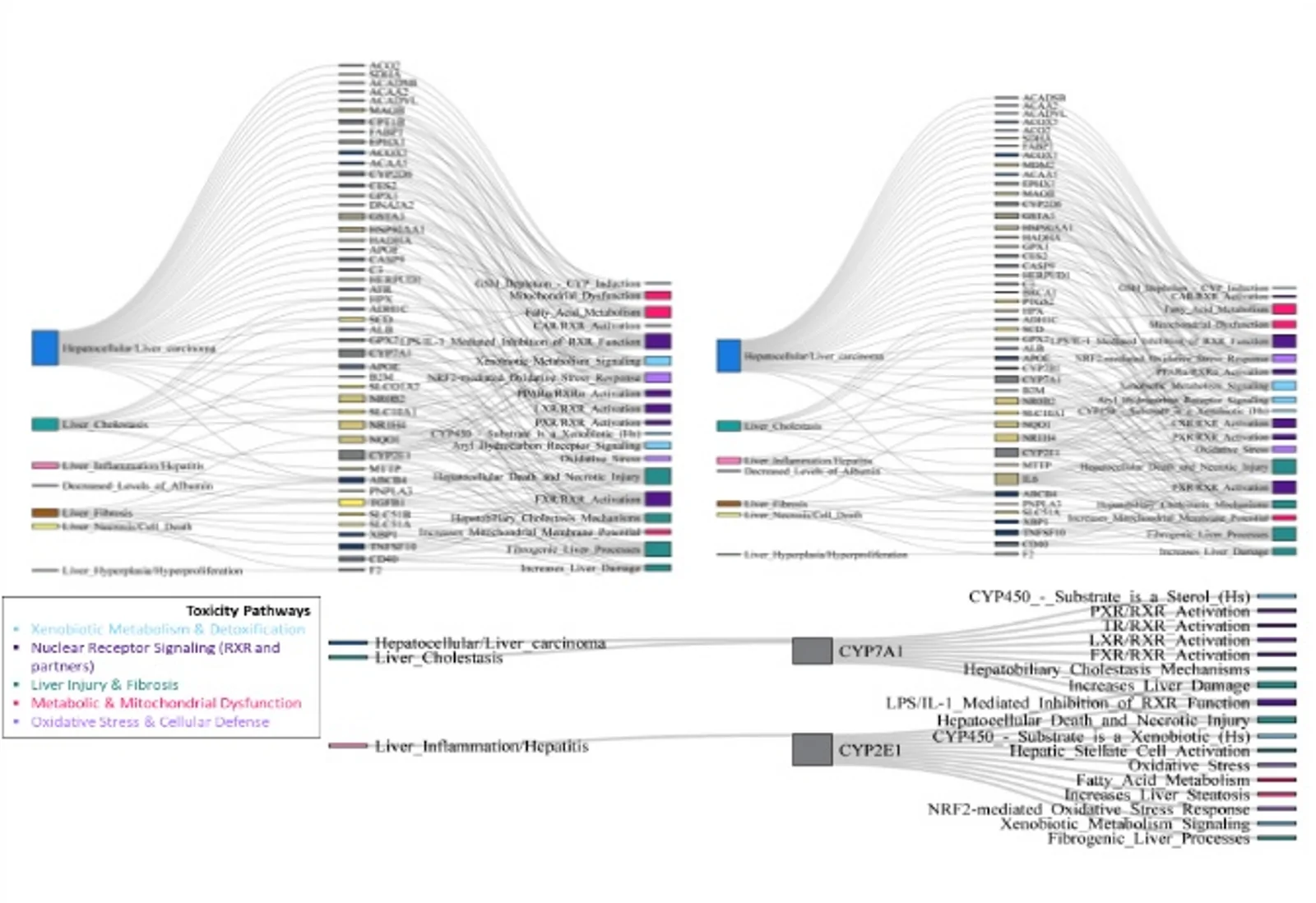
Flow diagrams of diseases/function annotation x differentially expressed genes (DEGs) x toxicity pathways predicted to be affected by each MC by the IPA. For each pathway—represented as nodes (left and right columns: diseases/function annotation and toxicity pathways, respectively)—the box height is scaled according to the number of genes involved

Flow diagrams were used to better illustrate the link between predicted toxicity pathways and disease/function annotations in the DEGs and pathways analysis (Fig. 5). In general, MC-LR and -LA were predicted to induce similar toxicity pathways. The underlying DEGs are associated with several liver pathologies, such as hepatocellular/liver carcinoma, cholestasis, and fibrosis. Although MC-RR also showed links between affected toxicity pathways and hepatocellular/liver carcinoma and cholestasis, these were based solely on two DEGs (CYP7A1, CYP2E1), indicating a much weaker effect than with -LR and -LA.

#### Potential miRNA-mRNA target interactions

The interaction between miRNAs and mRNAs is related to a key gene expression regulatory mechanism: ceRNAs (competing endogenous RNAs). In this study, ceRNA networks were constructed solely from experimentally validated miRNA-mRNA interactions, providing more biologically robust evidence for the networks’ predictions. According to the parameters used, no statistically significant negative correlations were found between DE miRNAs and DE mRNAs (Table SI). The absence of significant values may be related to the stringency of the criteria used for ceRNA network prediction. Considering that we opted to use only experimentally validated miRNA-mRNA interactions, this scenario might differ if we were to search for predicted miRNA targets for mRNAs. However, experimental validation is necessary to support the discussion of the ceRNA network as a key regulatory mechanism in the context analyzed. Another factor that may have limited the construction of statistically significant ceRNA networks is the scope of the DE mRNA search, which in this study was restricted to a panel of 384 genes. These results do not rule out the biological occurrence of ceRNA networks within the evaluated scenario; instead, they highlight a potential research window for expanding the investigative scope.

## Discussion

The responses to most markers (except PP2A, which showed a similar fold-inhibition rate) varied among the tested MCs, demonstrating toxicity response differences among congeners. Responses, particularly in expression-level changes in protein-coding genes and proliferative effects, show a very similar pattern for MC-LR and -LA, whereas the effects of -RR differ dramatically.

The gene expression changes observed in this study were marked by the downregulation of key genes associated with liver toxicity. Biales et al. (2020) reported a transcriptional response characterized mostly by upregulation in HepaRG cells exposed (2 h) to MC-LR and -RR, including genes downregulated in this study (*e.g.*, xenobiotic detoxification, antioxidant defense). Such differences are indeed expected for MCs, as varying degrees of liver damage at different exposure times and doses have been shown for this family of natural toxins. Ikumawoyi et al. (2024) showed that the 24 h MC-LR treatment in HepaRG cells captures a broader spectrum of protein changes associated with chronic disease-gene associations, especially those related to cancer.

With regard to potential liver adverse effects associated with the observed gene expression changes, it is known that liver injuries and diseases involve dysregulation of numerous genes and biological pathways (Au et al. 2011; Gan et al. 2025). Nuclear receptor (NR) signaling pathways, including those mediated by the retinoid X receptor alpha (RXRα), pregnane X receptor (PXR), and constitutive androstane receptor (CAR), are classically associated with chemical-induced liver toxicity (Vinken, 2024; Yang et al. 2024; Callewaert et al. 2025). In MC-treated cells, CAR/RXR, FXR/RXR, PXR/RXR, PPARα/RXRα, and aryl hydrocarbon receptor (AhR) pathways were enriched; however, most genes within the canonical pathways were downregulated, indicating pathway inhibition rather than the activation typically expected in response to chemical exposure (Sui et al. 2021; Bay and El-Masri, 2022; Lian et al. 2023). This can provide a mechanistic basis for the observed downregulation of CYP enzymes, as these NRs are key transcriptional regulators of phase I-II xenobiotic metabolism (Rakateli et al. 2023).

Comparable downregulating effects on CYP enzymes have been shown for OA, a marine natural toxin and potent inhibitor of PP2A. For OA, the effects were attributed to interactions with NF-ĸB/JAK/STAT and CAR pathways, in which PP2A plays a regulatory role (Wuerger et al. 2023, 2024). Despite similarities between MCs and OA in their CAR pathway-related regulatory mechanisms, this does not extend to the proposed NF-κB-mediated mechanism, which involves inflammatory response. *IL6* and *TNF*, key cytokines in this pathway, showed variable expression among the tested MCs in both magnitude and direction, despite comparable PP2A inhibition, suggesting the involvement of PP2A-independent regulatory mechanisms. PP2A inhibition is regarded as the primary key event of MC toxicity (Bouaïcha et al. 2019); however, recent experimental evidence suggests that MC-LR may have molecular targets beyond protein phosphatase inhibition (Ikumawoyi et al. 2024), which will be discussed further.

Oxidative stress is involved in the pathogenesis of several liver injuries and diseases, such as cholestasis and cancer (Copple et al. 2010; Liang et al. 2025). Genes related to antioxidant defense, such as *CAT* and *GPX2*, considered the first line of defense against oxidative stress (Ighodaro and Akinloye 2018), were strongly downregulated by MC-LR and -LA, suggesting that the transcriptional response may reflect oxidative stress. In particular, *GPX2* is associated with the glutathione defense system, which is recognized as an important component of detoxification and antioxidant response to MCs. Reactive oxygen species (ROS) generation in hepatocytes has been reported in MCs, and oxidative stress has been proposed as a mechanism underlying the in vivo tumor promotion activity of MC-LR (Zegura 2016; Lei et al. 2019). The role of MCs in hepatocarcinogenesis remains an open question due to limited in vivo carcinogenicity data for members of the MC family.

A recently published read-across study integrated multiple lines of evidence into a weight-of-evidence (WoE) approach to support the hypothesis of structural similarity and a shared mode of action (tumor promotion activity) for MCs. However, considering the quantitative in vitro differences (acute toxicity potency and OATP activity) and those related to cellular uptake and detoxification efficiency, this study suggested that different congeners could exhibit variable tumor promotion activity (*i.e.*, higher MC-LR and lower -RR tumor-promoting activity) (Pestana et al. 2025). The present in vitro data are consistent with the read-across study, as the strongest correlation was observed between the transcriptional responses to MC-LR and -LA and hepatocellular/liver carcinoma, whereas the corresponding correlation for -RR was very weak.

Several genes described as tumor suppressors in liver cancer, as well as markers associated with poor prognosis in HCC (e.g., *NR1H4*, *SLC10A1*, *CYP2C9*, and *CYP1A2*) (Yang et al. 2007; Huang et al. 2022; Ye et al. 2025) were significantly downregulated following exposure to MC-LR and -LA, but not to -RR. In addition, some of the most upregulated genes in MC-LR and -LA were *MKI67* (encoding Ki-67) and *RAD51*. Both genes are strongly associated with increased cell proliferation and poor clinical outcomes when overexpressed and are therefore widely used as HCC markers of tumor aggressiveness, proliferative rate, and prognosis (Takahashi et al. 2024; Jagtap 2025). These data are consistent with the significant increase in cell proliferation observed with MC-LR and -LA, further supporting their potential, tumor-promoting effects, in contrast to -RR.

Still, regarding hepatocarcinogenesis, the miRNA expression pattern was not consistently conserved across all three MCs. miRNAs are part of post-transcriptional regulation, and recent studies highlight their potential as markers of the early stages of carcinogenesis (Kohlhapp et al. 2015; Morishita et al. 2021). Although miRNA-mRNA target interactions were not sufficient to identify regulatory networks, likely due to the small number of target protein-coding genes (*i.e.*, 384 out of 21,616 in the human genome), the three MCs affected the expression of miRNAs associated with carcinogenesis. The MC-LA exerted the greatest effect on miRNA followed by -LR and -RR, and all three downregulated miRNA-219-5p. Low expression of this miRNA is found in HCC samples and is associated with larger tumor size and shorter overall survival time. Conversely, upregulation of miRNA-219-5p can inhibit cell proliferation and migration in vitro (Huang et al. 2012; Ting et al. 2020). miRNA-192-5p suppression (downregulated in MC-LA) is associated with HCC malignancy and has been implicated in HCC development (Gu et al. 2019; Yin et al. 2022).

Liver cholestasis, reported in vivo for MC-LR and supported by clinical evidence from a fatal MC intoxication event in dialysis patients (Azevedo et al. 2002; Yan et al.  2020), was also captured in the gene expression data. Cholestasis is a pathological condition characterized by impaired bile formation, excretion, or secretion, leading to bile acid accumulation and liver damage. AOPs have been defined for chemical-induced cholestasis (van Ertvelde et al. 2023). The transcriptional response associated with this liver injury was observed for all MCs, but with the highest probabilities identified for MC-LR and -LA. With respect to the mechanism underlying MC-induced cholestasis, the most plausible is inhibition of hepatobiliary transporters (e.g., NTCP, encoded by *SLC10A1* and downregulated by all MCs, but significantly only in MC-LR and -LA) and oxidative stress, ultimately leading to cholestasis. The inhibitory effect of MC-LR on NTCP and BSEP has also been demonstrated using a 3D HepaRG model (Chowdhury et al. 2024). In cholestasis, oxidative stress can also have downstream effects on cell death, with apoptosis as the preferred type in chemical-induced cholestasis, although necrosis can also occur (van Ertvelde et al. 2023). The downregulation of several caspases, including caspases 3 and 7, the major executioner caspases in apoptosis, suggests that cell death may occur predominantly via an alternative mechanism rather than apoptosis in this study. Necrosis, necroptosis, and ferroptosis are other forms of cell death reported to occur in response to MC-LR, both in vivo and in vitro (Zhu et al. 2023; Chowdhury et al. 2024). In particular, in ferroptosis – a type of oxidative cell death characterized by extensive lipid peroxidation – decreased GPX4 activity has been identified as a typical marker of this process (Chen et al. 2022). *GPX4* was significantly downregulated in MC-LR and -LA. Emerging evidence supports a role for ferroptosis in liver diseases, including cholestasis (Chen et al. 2022; Zhou et al. 2025).

Collectively, these findings indicate that MC-LR and -LA exert greater hepatotoxicity and provide stronger evidence of potential tumor promoting effects than -RR. Differences in biological responses among MC congeners have been associated with variations in toxicokinetics and interactions with protein phosphatases, particularly PP2A (Bouaïcha et al. 2019), although no clear consensus has been reached. In terms of PP2A, this protein phosphatase is recognized as a critical regulator of metabolic processes in hepatic cells, controlling post-translational modifications and cell cycle progression (Takumi et al. 2010; Tzima et al. 2017). Its inhibition adversely affects human health by disrupting multiple biological processes (Sun et al. 2014; Sallandt et al., 2025; Wuerger et al. 2024) and may therefore be a plausible regulator of MCs’ toxicity, as proposed. Furthermore, there is consensus that congeners vary in their potency to inhibit PP2A activity, although the magnitude of this variation is relatively modest (WHO, 2020). Values from the recombinant human PP2A catalytic subunit assay available for several congeners varied by no more than tenfold (Yang et al. 1999). This study revealed marked differences in biological responses among the three congeners despite comparable levels of PP2A inhibition under identical experimental conditions, suggesting that PP2A is unlikely to be the exclusive regulator of MC hepatotoxicity.

Differences in toxicokinetics (*e.g.*, cellular uptake and detoxication efficiency) have also been proposed to influence variations in toxicity responses among MCs, both in vivo and in vitro (Bouaïcha et al. 2019). Affinities for OATPs, membrane proteins that mediate the uptake of MCs into liver cells, vary among congeners. MC-LR and -LA exhibit higher affinity for OATP1B1, while -RR shows higher affinity for OATP1B3 (Chowdhury et al. 2024). In HepaRG cells, OATP1B1 is expressed at slightly higher levels than OATP1B3 (Chowdhury et al. 2024). This may facilitate the more rapid transport of MC-LR and -LA into HepaRG cells, potentially leading to different intracellular concentrations among the three congeners. Detoxification efficiency also varies with congener hydrophilicity, with MC-RR showing the highest detoxification capacity (Santori et al. 2020). Moreover, a covalent binding threshold for MC-LR to PP2A in HepaRG cells has recently been demonstrated, and its potential interactions with other kinases have also been reported (Ikumawoyi et al. 2024). Among the perturbed kinases identified in that study, upregulation of CHEK1 (a serine/threonine kinase involved in cell cycle regulation and implicated in carcinogenesis) was confirmed in the present study, reaching significance only for MC-LR and -LA. Overall, toxicokinetic differences, combined with PP2A binding thresholds, may enable MC-LR and -LA, but not -RR, to interact with other regulatory proteins, leading to differential responses in human hepatocytes.

Furthermore, the highly similar effect patterns of MC-LR and -LA, in contrast to -RR, raise the question of which structural differences may underlie their distinct toxicological profiles. MCs are very structurally similar, differing primarily in positions 2 and 4, which have been used to explain differences in OATP affinity and PP2A interactions. At position 4, MC-LR and -RR share the same amino acid, while -LA contains L-Ala. At position 2, MC-LR and -LA contain L-Leu, while -RR contains L-Arg (Bouaïcha et al. 2019). The positively charged Arg residue is associated with reduced potency, likely due to impaired cellular transport (Niedermeyer et al. 2014). The comparable gene expression and proliferative responses induced by MC-LR and -LA, but not -RR, support a central role for position 2 in modulating biological activity. However, further computational and in vitro studies with additional congeners are needed to clarify these structure–activity relationships.

## Conclusion

The tested MC congeners can induce hepatotoxicity and exhibit potential tumor promoting effects; however, the magnitude of these effects varies among congeners, reinforcing the concept of congener-specific toxicity within the MC family. Moreover, the present findings suggest that PP2A-independent mechanisms, rather than PP2A inhibition alone, account for most congener-specific differences in toxicity. Identifying the master regulators underlying MC-induced effects requires a comprehensive characterization of gene-expression changes, *i.e.*, transcriptomic data from key congeners. The in vitro approach employed in this study advances the understanding of toxicity mechanisms within the MC family by capturing in vivo-relevant adverse responses, particularly for MC-LR, for which most data are available. Consequently, this in vitro approach shows potential in toxicity studies of MC congeners and supports the advancement of risk assessment for this group of natural toxins.

## Supplementary Information

Below is the link to the electronic supplementary material.

Supplementary Material 1

## Data Availability

Data available on request from the authors.
