# Supplementary material for: Mechanistic investigation of the hepatotoxicity and potential carcinogenicity of microcystin-LR, -LA, and -RR in the HepaRG cell line model
Source: Arch Toxicol. 2026 Jun 8;100(10):4763–75. doi: 10.1007/s00204-026-04458-0 (PMC13593803; doi:10.1007/s00204-026-04458-0)
Supplement: Supplementary file 1 [file 204_2026_4458_MOESM1_ESM.docx]

Mechanistic investigation of the hepatotoxicity and potential carcinogenicity of Microcystin-LR, -LA, and -RR in the HepaRG cell line model

Enzo Zini Moreira Silva^a,b^, Sahra Kiessig^b^, Ilinca Suciu^b^, Carolina Mathias^a^, Marize de Lourdes Marzo-Solano^b^, Cynthia Bomfim Pestana^a^, Philip Marx-Stoelting^c,*^, Daniela Morais Leme^a,b^

^a^ Department of Genetics, Federal University of Paraná (UFPR), Curitiba, PR, Brazil.

^b^ German Federal Institute for Risk Assessment (BfR), Berlin, Germany Corresponding author: [Philip.Marx-Stoelting@bfr.bund.de](mailto:Philip.Marx-Stoelting@bfr.bund.de)

Figure S1. Cell viability data for the tested MCs after 24 h of exposure in the HepaRG cell line, using WST-1 and Neutral Red Uptake (NRU) assays. The data are expressed in mean ± SD of cell viability related to the negative control. The dashed line represents the control viability.

Figure S2. Principal Component Analysis (PCA) of mRNA dataset containing negative control (Control) and tested MCs. PC1 and PC2 were the components most representative of the variance.

Figure S3. Scatter plots of mRNA and miRNA tested dataset comparing each treatment. R^2^ was defined by linear regression

Figure S4. Heatmap of the mRNA dataset using average fold changes for each condition (relative to the control). Unsupervised hierarchical clustering of genes was done using Euclidean distance and complete linkage. * = p < 0.05 relative to the negative control.

Figure S5. Heatmap of the mRNA dataset, where each column represents an individual replicate. For each gene, the expression data were scaled across the tested samples and represent Z-scores.

Figure S6. Heatmap of the miRNA dataset, where each column represents an individual replicate. For each gene, the expression data was scaled across the tested samples and represents Z-scores.

Table SI. miRNA and mRNA expression correlation. Spearman r = Spearman rank correlation

|  | MC-LR |  | |
| --- | --- | --- | --- |
| miRNA | mRNA | spearman r | p-value |
| hsa-miR-18a-3p | GPD1 | -0,5 | >0,9999 |
|  | CES2 | -0,5 | >0,9999 |
|  | TXNIP | -0,5 | >0,9999 |
|  | SCD | -0,5 | >0,9999 |
|  | EHHADH | -0,5 | >0,9999 |
|  | SDHA | -0,5 | >0,9999 |
| MC-LA | | | |
| miRNA | mRNA | spearman r | p-value |
| hsa-miR-383-5p | ACO2 | -0,5 | >0,9999 |
|  | SCD | -0,5 | >0,9999 |
|  | SERPINA3 | -0,5 | >0,9999 |
| miRNA | mRNA | spearman r | p-value |
| hsa-miR-195-5p | ASNS | 0,5 | >0,9999 |
|  | CD44 | 0,5 | >0,9999 |
|  | CHEK1 | 0,5 | >0,9999 |
| miRNA | mRNA | spearman r | p-value |
| hsa-miR-192-5p | BRCA1 | 0,5 | >0,9999 |
|  | BRCA2 | 0,5 | >0,9999 |
|  | MDM2 | 0,5 | >0,9999 |
|  | MKI67 | 0,5 | >0,9999 |
|  | RAD51 | 0,5 | >0,9999 |
| miRNA | mRNA | spearman r | p-value |
| hsa-miR-200c-3p | CD44 | 0,866 | 0,6667 |
|  | CHEK1 | 0,866 | 0,6667 |
|  | MDM2 | 0,866 | 0,6667 |
|  | NQO1 | 0,866 | 0,6667 |
|  | PTGS2 | 0,866 | 0,6667 |
| miRNA | mRNA | spearman r | p-value |
| hsa-miR-135a-5p | MDM2 | 0,5 | >0,9999 |
|  | BRCA1 | 0,5 | >0,9999 |
| miRNA | mRNA | spearman r | p-value |
| hsa-miR-195a-5p | GALNT5 | 0,5 | >0,9999 |
| miRNA | mRNA | spearman r | p-value |
| hsa-miR-216a-5p | CD44 | 0,5 | >0,9999 |
| MC-RR | | | |
| miRNA | mRNA | spearman r | p-value |
| hsa-miR-7-5p | CPT1B | 1 | 0,3333 |
|  | ATP6V1G2 | 1 | 0,3333 |
|  | CD4 | 0,5 | >0,9999 |
| miRNA | mRNA | spearman r | p-value |
| hsa-miR-135b-5p | CPT1B | 1 | 0,3333 |
